# Cancer in a Drop: Liquid biopsy insights from AACR 2025

**DOI:** 10.1016/j.jlb.2025.100304

**Published:** 2025-06-10

**Authors:** Shivahamy Maheswaran, Roberto Borea, Diego de Miguel Perez, Pasquale Pisapia, Nadia Ghazali, Canio Martinelli, Pavel Stejskal, Carolina Reduzzi

**Affiliations:** a Department of Medicine, University of Massachusetts Chan Medical School, Worcester, MA, USA; b Division of Medical Oncology, Department of Internal Medicine, The Ohio State University Comprehensive Cancer Center, College of Medicine, The Ohio State University Wexner Medical Center, Columbus, OH, USA; c Department of Internal Medicine and Medical Sciences (DiMI), School of Medicine, University of Genova, Genova, Italy; d Department of Public Health, University Federico II of Naples, Naples, Italy; e Division of Medical Oncology and Hematology, Princess Margaret Cancer Centre (PMCC), University Health Network (UHN), Toronto, ON, Canada; f University of Toronto, Toronto, ON, Canada; g Sbarro Health Research Organization, Temple University, Philadelphia, PA, USA; h Institute of Molecular and Translational Medicine, Faculty of Medicine and Dentistry, Czech Advanced Technology and Research Institute, Palacky University and University Hospital in Olomouc, Czech Republic; i Liquid Biopsy Platform, Department of Medicine, Weill Cornell Medicine, Englander Institute for Precision Medicine, New York Presbyterian Hospital, New York, NY 10021, USA; j International Society of Liquid Biopsy (ISLB) Young Committee, Spain

## 1 Introduction

The American Association of Cancer Research (AACR) Annual Meeting is an internationally renowned conference that consolidates and reports the latest breakthroughs in basic, translational, clinical, and epidemiological sciences. This editorial, written on behalf of the International Society of Liquid Biopsy (ISLB) Young Committee, highlights the most recent research efforts presented at the AACR Annual Meeting 2025, focusing on liquid biopsy (LB) methods and applications. As our understanding of LB continues to expand, its role along the cancer care continuum becomes increasingly apparent. LB played a key role in this year’s AACR Annual Meeting with four dedicated oral sessions, over 30 oral presentations, and six poster sessions. While most presented studies focused on the analysis of cell-free DNA (cfDNA), several were dedicated to other analytes such as circulating tumor cells (CTCs), with a dedicated minisymposium, and extracellular vesicles (EVs), both in blood and in a non-blood setting. Here, we report a selection of the most relevant presented abstracts, with an emphasis on three main areas of LB application: i) screening, detection and diagnosis, ii) minimal residual disease (MRD) monitoring, iii) prediction and prognostication (Table 1).

**Table 1:** Summary of notable oral presentations on liquid biopsy at AACR 2025.

Topic	Title [ref]	Main findings
Diagnostic/secreening/detection		
Multi-cancer early detection platforms	The vanguard study: An NCI cancer screening research network feasibility study [1]	The study will stablish the feasibility and equitable recruitment strategies for future large-scale randomized controlled trials of multi-cancer detection assays, incorporating unique design elements like remote consenting and tailored diagnostic workups
Multi-cancer early detection platforms	Pre-diagnostic circulating proteins and breast cancer risk in the European Prospective Investigation into Cancer and Nutrition [2]	This study identified 19 proteins associated with premenopausal and three with postmenopausal breast cancer risk, suggesting novel pathways and potential biomarkers that vary by menopausal status and require replication in other studies.
Early detection in inflammation/cancer continuum	Cell-free DNA fragmentomes enable early identification of liver cirrhosis to facilitate cancer surveillance [5]	Cell-free DNA fragmentome analysis effectively identifies liver cirrhosis with high accuracy, outperforming existing fibrosis indices, which could facilitate earlier diagnosis and improved hepatocellular carcinoma surveillance
Early detection in inflammation/cancer continuum	Multiomic functional biomarkers for predicting the transition from inflammation to cancer [6]	This multi-omics study validated a 27-plasma biomarker panel and CHIP mutations that effectively predicted cancer development in smokers with cardiovascular disease and Li-Fraumeni syndrome, identifying distinct high-risk subgroups potentially amenable to targeted interceptive measures
Minimal Residual Disease		
New technology	MUTE-Seq: An ultrasensitive method for detecting low-frequency mutations in cfDNA with engineered advanced-fidelity FnCas9 [12]	A novel method leveraging a highly precise FnCas9-AF2 variant to selectively eliminate wild-type DNA, thereby enabling highly sensitive detection of low-frequency cancer-associated mutations
LB use in clinical trials	Comparison of ctDNA detection methods for monitoring minimal residual disease in patients with bladder cancer: Insights from the TOMBOLA Trial [10]	A comparison of droplet digital PCR (ddPCR) and whole-genome sequencing (WGS) for circulating tumor DNA (ctDNA) detection in 1,282 paired plasma samples revealed an 82.9 % concordance, with ddPCR showing higher sensitivity in low tumor fraction samples but both methods demonstrating comparable predictive power for recurrence-free survival and similar lead times over imaging
Prediction and Prognostication		
LB use in clinical trials	Combined tissue and liquid biopsy improves outcomes in advanced solid tumors: an exploratory analysis of the ROME trial [22]	Despite only 49 % concordance between tissue and liquid biopsies in detecting actionable alterations, combining both modalities significantly increased overall detection of actionable alterations and led to improved survival outcomes in patients receiving tailored therapy, highlighting the importance of integrated approaches in precision oncology
LB use in clinical trials	Morphological evaluation of chromosomal instability in circulating tumor cells and prediction of taxane resistance in metastatic prostate cancer: a prespecified sub-analysis of the CARD trial [20]	High baseline circulating tumor cell (CTC) counts, particularly those exhibiting chromosomal instability (CTC-CIN), were significantly associated with worse overall survival and suggested that low CTC-CIN at baseline could predict a greater benefit from cabazitaxel treatment
LB use in clinical trials	Association of baseline ctDNA EGFR mutation detection with clinical outcome in the phase II RAMOSE trial assessing ramucirumab plus osimertinib versus osimertinib in EGFR mutant non-small cell lung cancer [19]	Baseline detection of EGFR mutations in plasma, particularly at a variant allele frequency greater than 0.5 %, was prognostic for significantly shorter PFS and OS in patients with EGFR-mutant NSCLC treated with osimertinib, suggesting its potential use for patient stratification in future studies

## 2 Detection/screening/diagnosis

This year’s AACR Annual Meeting highlighted key progress in the use of LB approaches for cancer screening, risk stratification, and diagnosis. Numerous large-scale efforts investigated the clinical efficacy of multi-cancer early detection (MCED) platforms. The Vanguard Study, part of the NCI Cancer Screening Research Network, demonstrated the feasibility of implementing MCED tests in real-world settings. Enrolling over 6,200 participants, the study confirmed high adherence and operational viability across diverse populations [1]. A proteomic analysis was conducted within the European Prospective Investigation into Cancer and Nutrition (EPIC) cohort to identify circulating plasma proteins associated with breast cancer (BC) risk. Nineteen proteins were associated with premenopausal BC risk, and three (LEG1, CST6, SAR1B) with postmenopausal risk. Heterogeneity tests showed that 20 proteins were differentially associated between BC risk in premenopausal and postmenopausal women [2]. Forouzmand et al. developed a novel algorithm, from a plasma-based sequencing platform, capable of predicting the cancer signal of origin (CSO) of 12 tumor types (bladder, breast, colorectal, esophageal, kidney, liver, lung, ovarian, pancreatic, prostate, stomach, and uterine/endometrial) using cfDNA methylation signatures. The CSO classifier achieved 88.2 % top prediction accuracy (93.6 % when considering the top two predictions) [3]. Another MCED test using a hybrid-capture methylation assay was evaluated, which demonstrated high specificity (98.5 %) and overall sensitivity (59.7 %). There was high sensitivity in late-stage tumors (84.2 %), cancers without average risk standard-of-care screening (73 %) and aggressive cancers such as pancreatic, liver, and esophageal carcinomas (74 %) [4]. Multiple studies assess the role of LB in early cancer detection under chronic inflammatory clinical settings. In liver cancer, cfDNA fragmentomics distinguished cirrhosis and hepatocellular carcinoma from healthy states with high accuracy with an area under the curve (AUC) of 0.92 in a 724-person cohort. These low-coverage whole genomic sequencing-based approaches could enable earlier intervention in high-risk populations [5]. Another group, using a multi-omic analysis in smokers with cardiovascular disease, and developed and validated a set of 27 plasma biomarkers predicting time to cancer diagnosis across various smoking-associated cancers [6]. LB is also being harnessed to ameliorate diagnostic accuracy of existing tissue-based modalities. One study presented a methylation-based deconvolution model capable of quantifying proportions of lung cancer histology subtypes of lung adenocarcinoma (LUAD), lung squamous cell carcinoma (LSCC), and small cell lung carcinoma (SCLC) within a single blood sample. This model had an accuracy of 85.1 % with the ability to detect down to tumor fraction of 0.1 %, with accuracies for LUAD (90.4 %), LSCC (72.4 %) and SCLC (63.5 %). The model was also able to detect minor subclonal populations as low as 0.1 % [7].

## 3 Minimal residual disease

MRD detection through LB remains an ongoing area of investigation, and its clinical value was explored in various studies presented at AACR. In colorectal cancer, the VICTORI study enrolled 160 patients. Circulating tumor DNA (ctDNA) analysis was performed pre- and post-surgery using the neXT Personal MRD detection assay, yielding 94.3 % ctDNA positivity in treatment-naive patients and 72.4 % in patients with radiologically evident disease who received neoadjuvant therapy. Crucially, 87 % of recurrences were preceded by ctDNA positivity, whereas no ctDNA-negative patient relapsed [8]. In bladder cancer, uRARE-seq, a high-throughput cell-free RNA (cfRNA)-based workflow, was used to analyze urine samples for MRD assessment in 36 patients. The assay showed 94 % sensitivity (LOD_95_ = 0.05 %), higher in patients with more advanced stages—and was associated with shorter high-grade recurrence-free survival both before and after Bacillus Calmette–Guérin (BCG) therapy [9]. In the TOMBOLA trial, droplet digital polymerase chain reaction (ddPCR) and whole genome sequencing (WGS) analysis was performed on plasma collected from bladder cancer patients during neoadjuvant chemotherapy, at radical cystectomy (RC), post-RC, and during immunotherapy. ctDNA detection in plasma using ddPCR and WGS methods showed overall high concordance; however, 12.9 % of the samples were positive only by ddPCR, indicating some discordance between methods [10]. Two studies on lung cancer were presented. The first, which was concomitantly published on Cancer Discovery, used the CIRI-LCRT model, a novel composite score integrating radiomic and pathological features from post-chemoradiation computed tomography scans with serial ctDNA measurements. In a cohort of 474 patients with non-small cell lung cancer (NSCLC) treated with chemo-radiation, the composite score predicted progression a median of 2–3 months ahead of conventional post-treatment MRD assays [11]. The second used the Mutation tagging by CRISPR-based Ultra-precise Targeted Elimination in Sequencing (MUTE-Seq) method, which was adopted for the enrichment of mutant DNA through the exclusive cleavage of perfectly matched wild-type DNA, allowing for sensitive detection of low-frequency cancer-associated mutant-alleles. The MUTE-Seq assay was used for MRD evaluation in patients with NSCLC and pancreatic cancer and demonstrated a significant improvement in the sensitivity of simultaneous mutant detection [12].

## 4 Prediction/prognostication

Collective evidence from presentations, spanning from pediatric tumors to adult solid malignancies, underscores the breadth of LB for prognosis and prediction of cancer. In neuroblastoma, plasma EV concentration and nucleolin expression were found higher in onset in high-risk patients, suggesting its potential as prognostic biomarker for stratifying pediatric patients and informing timely therapy intensification [13]. Serum proteomics showed importance in trastuzumab deruxtecan–treated patients, where elevated inflammatory proteins, notably CXCL11, were associated with impending interstitial lung disease/pneumonitis, hinting at a future role for serum profiling in proactive toxicity management [14]. Similarly, a novel fluorescent dCFPyL probe offered an alternative lens for metastatic prostate cancer, enabling detection of both CTCs and immune cells harboring cancer-derived EVs, which may refine risk classification and surveillance strategies [15]. In SCLC, cfDNA fragmentome analysis aligned with flow cytometry of peripheral blood immune subsets, accurately capturing neuroendocrine differentiation and anticipating immunotherapy outcomes [16]. Extending fragmentome-based approaches, a tumor-naive algorithm measuring cfDNA integrity also demonstrated robust predictive power for immunotherapy response in metastatic NSCLC, outperforming mutation-based methods for early detection of treatment benefit [17]. In head and neck cancer, personalized cfDNA assays showed matching methylated regions in primary tumors and node metastasis, demonstrating capabilities for identifying occult nodal metastases and guiding more conservative surgical approaches [18]. Meanwhile, baseline epidermal growth factor receptor (*EGFR*) mutation detection in ctDNA correlated with worse progression-free and overall survival in EGFR-mutant NSCLC, suggesting that patients with higher variant-allele frequencies may benefit from intensified strategies or closer monitoring [19]. The morphological evaluation of chromosomal instability in CTCs from metastatic prostate cancer further highlighted the value of LB in predicting taxane resistance, potentially sparing patients from inefficient regimens [20]. Finally, an exploratory analysis from the ROME trial was presented, showing a concordance between tissue and liquid biopsy alteration detection of 49 % [21,22]. Most discordant cases (43.3 %) were due to differences in the detection of molecular alterations, suggesting high intra-tumoral heterogeneity in these tumors. Interestingly, the survival benefit observed in the overall population treated with tailored therapy vs standard-of-care (median OS 11.05 vs. 7.70 months) was higher in patients with concordant alteration detection in tissue and liquid (11.05 months), followed by the tissue-only group (9.93 months), and the liquid-only group (4.05 months). Similar results were reported for PFS. Overall, LB testing not only allowed to increase alteration detection, but the combination with tissue analysis enabled the identification of a subgroup of patients with the highest survival benefit from tailored treatment, although these results need to be confirmed in future studies. One study investigated the detection of CTCs in the cerebrospinal fluid (CSF) of patients with leptomeningeal disease from metastatic breast cancer. This method appears to be of increased sensitivity (70 % detection rate in mock samples) compared to standard CSF cytology [23].

## 5 Conclusions

LB has gained traction as an essential tool in optimizing cancer care, and presented works at the 2025 AACR annual meeting showcased the breadth of its potential clinical impact. Screening, diagnosis and risk stratification were prominently featured with a notable emphasis on multi-cancer detection systems. Multiple studies identified biomarkers of early malignant transformation in the inflammation–cancer continuum. However, there is still scarce work on extending the therapeutic reach of LB and increasing access for high-risk populations outside large academic institutions. LB also had increased representation in clinical trials, especially under predictive and prognostic contexts. New studies comparing tissue and LB for alteration detection showed the importance of integrating both, to reach a higher detection of actionable mutations, important for improving treatment selection. Nonetheless, validating the clinical utility of LB use remains an ongoing issue, and more prospective clinical trials that employ LB in its study design are needed before these new applications can be used in clinical practice. As the LB field matures with more clinical validation studies, integrating proteomic, genomic, and cellular analyses will likely usher in a new standard of precision oncology care.

## Ethical approval

No ethical approvals or patient consent were necessary for the study.

## Declaration of competing interest

The authors declare the following financial interests/personal relationships which may be considered as potential competing interests: Carolina Reduzzi reports a relationship with 10.13039/100016273Menarini Silicon Biosystems Inc that includes: funding grants. Carolina Reduzzi reports a relationship with 10.13039/100017191ANGLE plc that includes: non-financial support. Carolina Reduzzi reports a relationship with Tethis spa that includes: non-financial support. If there are other authors, they declare that they have no known competing financial interests or personal relationships that could have appeared to influence the work reported in this paper.
